# Allograft or autograft in skeletally immature anterior cruciate ligament reconstruction: a prospective evaluation using both partial and complete transphyseal techniques

**DOI:** 10.1186/s13018-019-1128-7

**Published:** 2019-03-21

**Authors:** Mohammad Razi, Amin Moradi, Afsane Safarcherati, Alireza Askari, Peyman Arasteh, Elaheh Ziaei Ziabari, Haleh Dadgostar

**Affiliations:** 1 Department of Orthopedic Surgery, Rasoul Akram Hospital, Iran University of Medical Sciences, Tehran, Iran; 2 Department of Orthopaedic Surgery, Atieh private Hospital, Tehran, Iran; 3 Sports Medicine Department, Rasoul Akram Hospital, Iran University of Medical Sciences, Tehran, Iran; 4 Bone and Joint Reconstruction Research Center, Shafa Orthopedic Hospital, Iran University of Medical Sciences, Tehran, Iran; 5 Department of Orthopedics, School of Medicine, Shiraz University of Medical Sciences, Shiraz, Iran; 6 Noncommunicable Diseases Research Center, Fasa University of Medical Sciences, Fasa, Iran; 7 Department of MPH, Shiraz University of Medical Sciences, Shiraz, Iran; 8 Iran University of Science and Technology, Tehran, Iran

**Keywords:** Autograft, Allograft, Anterior cruciate ligament, Reconstruction, Adolescent

## Abstract

**Objective:**

We compared autografts and allograft using partial and complete transphyseal anterior cruciate ligament (ACL) reconstruction techniques among skeletally immature individuals.

**Methods:**

Male and females younger than 18 and 16 years old, respectively, diagnosed with ACL tear from April 2006 to March 2012 entered the study. One group had four-strand hamstring autograft, and the other had tibialis posterior allograft reconstruction. Those who had allografts either had hyper-laxity or recurvatum.

**Results:**

Achieved mean (± SD) 2000 International Knee Documentation Committee subjective score was not statistically different (*P* = 0.385) between allograft (*n* = 13) (84.3 ± 3.2) and autograft groups (*n* = 18) (85.6 ± 4.4). Mean Knee injury and Osteoarthritis Outcome Score (KOOS) subscale Knee-Related Quality of Life at 2 years was 78.0 ± 7.2 and 75 ± 7.4 for allograft and autograft groups, respectively (*p* = 0.261). Mean 2-year KOOS subscale Sports and Recreation was 82.1 ± 5.8 and 84.8 ± 6.6 for allograft and autograft groups, respectively (*p* = 0.244).

No patient reported instability, giving way, or locking of the knee. Pivot shift test was negative in all patients; however, a minor positive Lachman test was found in six cases (46%) within the allograft group and seven cases (39%) in the autograft group. One postoperative septic arthritis was documented in the autograft group.

**Conclusion:**

Considering existing concern that joint laxity and recurvatum are among the precursors of non-contact ACL injury in adolescents, bone-patellar-bone autografts are not applicable in this age group because of the open physis; furthermore, considering that hamstring autografts are insufficient (size thickness and stretchability), we recommend soft tissue allografts for ACL reconstruction in skeletally immature patients.

## Introduction

Anterior cruciate ligament (ACL) injuries have become more prevalent in the skeletally immature patient over the past 20 years [1]. Potential factors include increased numbers of athletes, continuous sports participation, and increased focus on a single sports activity [2, 3].

Nowadays, most pediatric orthopedic surgeons recommend surgical reconstruction once the diagnosis of complete tear of ACL is confirmed [4]. The outcome of nonoperative treatment of ACL tears, compared to surgical treatment, in skeletally immature patients who return to sports is poor [5]. These children experience frequent instability and sports dysfunction. Prolonged instability leads to further meniscal injury and osteochondral damage resulting in early degenerative changes [3, 6].

The rate of successful ACL reconstructive surgery has been reported to be about 90% in restoring knee stability and patient satisfaction in the adult population [7, 8]; however, there is no consensus regarding the best method to treat an ACL tear in the pediatric athletes [9]. If surgery is chosen as the treatment modality, controversy exists regarding both graft and technique of choice [10]. Incomplete or partially ossified tibial spine is generally weaker to tensile stress rather than the ligament, so failure through the cancellous bone beneath the subchondral bone of the tibial spine is more common than mid-substance ACL tear [11]. So, in adolescents with mid-substance rupture of ACL, failure of reconstructive surgery is more likely attributable to weakness of collagen fibers and their connections. Therefore, the selection of allografts for ACL reconstruction for adolescents seems a logical choice. On the other hand, some recent literature has shown that the use of allografts in the adolescent athlete leads to an unacceptable failure rate [12, 13].

There is much debate regarding the size and tensile resistance of autografts in those who are considered to be skeletally immature [14, 15]. Moreover, soft tissue grafts have been shown to have minimal effects on growth [8, 9].

Selecting an autograft or an allograft for ACL reconstruction remains to be highly controversial among skeletally immature individuals. In this study, we compared two of the most commonly used grafts (autograft versus allograft) using both partial and complete transphyseal ACL reconstruction techniques among a sample of skeletally immature individuals.

## Materials and methods

### Study setting and patient selection

This is a prospective study conducted from April 2006 to March 2012. All male patients younger than 18 years old and female patients younger than 16 years old who referred to Hazrat Rasoul Hospital, affiliated to Iran University of Medical Sciences, Tehran, Iran, with a diagnosis of ACL tear for ACL reconstructions were included in the study.

Degree of maturation was evaluated according to the Tanner staging system based on external primary and secondary sex characteristics [16]; furthermore, status of physes, around the knee, was assessed using MRI (Tables 1).

**Table 1:** Tanner staging and ACL reconstruction specifics in the allograft and autograph groups

Type of graft	Tanner stage	Partial transphyseal ACLR	Complete transphyseal ACLR
Allograft, no. (%)	II–III	5 (41.6)	–
	IV	–	8 (42.1)
Autograft, no. (%)	II–III	7 (58.3)	–
	IV	–	11 (57.8)

*ACLR* anterior cruciate ligament reconstruction

Any individual who had a history of previous surgery in the knee, associated fractures, other major ligament injuries, or chondral lesions (grade III or VI Outerbridge) were excluded from the study. Furthermore, patients with Tanner stage 0 and 1 (pre-pubertal stage) and with Tanner stage 5 (closed physes in X-Ray) were also excluded from the study. Weight-bearing antero-posterior and lateral radiographs were obtained to assess the physes around the knee and to rule out tibial eminence fractures.

Complete ACL rupture was diagnosed based on clinical examination and was further confirmed by MRI prior to surgery. Once full range of motion was regained, one of the two types of ACL reconstruction techniques was performed on the patients based on Tanner stages. Patients with Tanner stages 2 and 3 (more than 1 year of growth remaining) underwent partial transphyseal ACL reconstruction. Patients with Tanner stage 4 (less than 1 year of growth remaining) underwent complete transphyseal ACL reconstruction.

Patients were categorized into two groups based on graft types. One group underwent ACL reconstruction with four-strand hamstring autografts (as group 1), and the other group underwent ACL reconstruction using tibialis posterior allografts (group 2). Those who had allografts were those who either had hyper laxity or recurvatum.

Surgical techniques and postoperative rehabilitation programs were similar in both groups. Moreover, all patients underwent reconstructive surgery by the same supervising attending surgeon in order to minimize any bias related to surgical techniques used.

### Procedures and grafting

For partial transphyseal ACL reconstruction, the patient was positioned supine on a fluoroscopy-compatible operating room table with a pneumatic tourniquet at the root of the thigh. A diagnostic arthroscopy was first performed through standard portals. Meniscal lesions that were likely to heal, stable to the palpating hook and smaller than 10 mm in size, were neglected and left in place. Unstable lesions were sutured with the inside-out, outside-in, or the all-inside techniques. Small and superficial chondral lesions were debrided. Complete and nonfunctional ACL ruptures were confirmed in direct view. The remnants of the ACL were preserved if possible. Some parts of the remnants that may have caused impingement were debrided.

In the autograft group, a 2- to 3-cm incision centered on the pes anserinus was made 2 cm medial to the anterior tibial tubercle. The semitendinosus tendon was harvested using a tendon stripper. Then, the tendon was folded into a four-strand graft and then placed under 20 lb of tension for 10 min. The goal graft diameter was 7 to 8 mm depending on the patient’s age and body mass.

In the allograft group, tibialis posterior allografts were used for the reconstruction. Cryo-preserved allografts (Tissue regeneration coop. (TRC), Yazd, Iran) were used in this study. Allograft tendon was transported to the hospital on dry ice under temperature-controlled conditions. For the thawing process, the graft was immersed in sterile saline warmed to 37 °C for 30 min, then placed under 20 lb of tension for 20 min. Similar to the autograft group, the ideal graft diameter was 7 to 8 mm depending on the patient’s age and body mass index.

Initially, the transepiphyseal femoral tunnel was approached. A 3-cm lateral incision was made at the lateral femoral condyle. The iliotibial tract was incised longitudinally, and the periosteum was stripped. Then, the ACL tibial jig was inserted through the anterolateral portal, and a guide pin was placed percutaneously through the lateral femoral condyle from outside in. The guide pin was directed into the notch under anteroposterior fluoroscopy, staying parallel and about 10 mm distal to the femoral growth plate. Moreover, it was kept perpendicular to the femur in the coronal plane and was visualized arthroscopically, entering into the intercondylar notch just at the center of the anatomic footprint of the ACL on the femur. In the lateral C-arm view, the guide wire crossed the lateral femoral condyle at a point that is one fourth of the distance from posterior to anterior along the Blumenstaat’s line. The femoral tunnel was reamed after the tibial tunnel guide pin was placed.

The tibial tunnel was placed centrally in the anatomic footprint of the ACL. A pin was inserted using ACL tibial jig at 50° and aimed at the residual ACL fibers at the ACL footprint. Impingement of the future graft on the notch was checked during gradual knee extension with the pin in place. The tibial tunnel was made using a proper drill bit, with a slow rotation speed to avoid overheating the physis.

Then, the graft was passed and fixed at the femoral side using a bioabsorbable interference screw (Smith & Nephew, London, UK). Femoral fixation was augmented by suturing the graft to the iliotibial band. At the tibial side, the graft fixation was done by one tendon staple.

Complete transphyseal ACL reconstruction, used in Tanner stage 4 patients, was performed similar to the partial transphyseal technique. However, in the complete transphyseal ACL reconstruction, the femoral tunnel crossed the physis of the femur. The femoral tunnel was created via the anteromedial arthroscopy portal approach. In the autograft group, the graft was fixed by the Endobutton (Smith & Nephew, London, UK) proximally and one bioabsorbable interference screw at the tibial side. Fixation of the allograft, at the femoral side, was done with both the Endobutton and the bioabsorbable interference screw. At the tibial side, the graft was fixed by the bioabsorbable interference screw and augmented by one staple.

### Postoperative care and follow-up

Rehabilitation programs were similar in all patients. Patients’ knees were placed in a simple knee immobilizer brace for 3 weeks, postoperatively. Quadriceps muscle contraction and straight-leg rises were started as soon as possible after surgery. Range of motion exercises, hamstring muscle exercises, while the patient was prone, and partial weight bearing with two crutches were started the day after surgery. At the end of the second month, proprioceptive exercises were started. Sports resumption was very gradual and was closely monitored by an independent orthopedic surgeon. Straight-line running was started at the fourth postoperative month. The practice of pivot and competitive sports was not allowed until the ninth postoperative month.

All patients were evaluated 3, 6, 12, and 24 months after surgery by an independent observer. The observer was blinded to the grouping of patients. Physical examination included objective assessment of length and alignment of lower extremities as well as range of motion and testing of knee instability with a manual Lachman and pivot shift test. All complications and operative revisions that occurred during a 24-month follow-up period were recorded. At 24 postoperative months, patients were reassessed according to two subscales (Knee-Related Quality of Life and Sports and Recreation) of the Persian-version of the Knee injury and Osteoarthritis Outcome Score (KOOS) [17] and according to the 2000 International Knee Documentation Committee (IKDC) Subjective Knee Evaluation Form [18]. Radiographic evaluation included weight-bearing antero-posterior and lateral alignment views for patients with obvious mal-alignment and shortening or with complaint of shortening or limping.

Laxity was assessed using the Beighton scale [19]. The Beighton score is among the most common and easy to perform techniques by which hypermobility is measured. It includes a 9-point scale. A higher number represents higher degree of laxity. We considered a score of 4 or more as hypermobility among our patients. The scale has been proven both reliable and valid among populations [20, 21].

### Statistical analysis

Data analysis was done using the SPSS software® for Windows®, version 16. For comparison of quantitative data with normal distribution between groups, the independent *t* test was used, and for comparison of quantitative data without normal distribution between groups, the Mann-Whitney test was used. For comparison of qualitative data, the chi-square and Fischer exact tests were used. Data are presented as means and standard deviations and median and interquartile range, where appropriate.

A *p* value of less than 0.05 was considered statistically significant.

## Results

Thirty-four cases entered the study during the 6-year study period. Among which, three patients were lost to follow-up. Tibialis posterior tendon allograft was used for ACL reconstruction in 13 patients. Eighteen patients underwent ACL reconstruction with semitendinosus autografts.

There were four bucket handle tears of the medial meniscus in the allograft group that were repaired using the inside-out technique. Two posterior flap tears of the lateral meniscus were seen that were managed by partial meniscectomy. One case (Tanner stage 4) had lateral collateral ligament augmentation with allograft due to moderate lateral knee instability.

In the autograft group, there were six bucket handle medial meniscal tears that were repaired using the inside-out technique. Furthermore, five posterior flap lateral meniscal tears were seen that were managed by partial meniscectomy. There was partial medial collateral ligament laxity in one case that was managed by medial reefing.

There was no deep infection in the allograft group. However, in the autograft group, one postoperative septic arthritis was documented that presented 8 days after ACL reconstruction, which was successfully eradicated after one stage arthroscopic irrigation and lavage and proper antibiotic therapy. In this case, the autogenous ACL graft appeared intact, so the graft was retained.

The achieved mean (± SD) 2000 IKDC subjective score did not have a statistically significant difference (*P* = 0.385) between the allograft (84.3 ± 3.2) and autograft groups (85.6 ± 4.4). Mean KOOS subscale Knee-Related Quality of Life at 2 years was 78.0 ± 7.2 and 75 ± 7.4 for the allograft and autograft groups, respectively, which was not statistically different between the two groups (*p* = 0.261). Mean 2-year KOOS subscale Sports and Recreation was 82.1 ± 5.8 in the allograft group and 84.8 ± 6.6 in the autograft group, which was not statistically significant (*p* = 0.244).

In both groups, no patient reported obvious instability, giving way, or locking of the knee. Pivot shift test was negative in all patients, but a minor positive Lachman test was found in six cases (46%) within the allograft group and seven cases (39%) in the autograft group (Table 2).

**Table 2:** Comparison of baseline and clinical characteristics between the allograft and autograft groups

Variables	Allograft (*n* = 13)	Autograft (n = 18)	*p* value
Sex, no. (%)			
Male	8 (61.5)	13 (72.2)	0.5302
Female	5 (38.5)	5 (27.8)	
Age, years	14.6 ± 0.89	15.0 ± 1.12	0.3161
Interval between injury to surgery, months	5.5 ± 2.90	4.2 ± 3.09	0.2413
Associated meniscus tear, no. (%)			
No tear	7 (53.8)	7 (38.9)	0.6372
Menisectomy	2 (15.4)	5 (27.8)	
Repair	4 (30.8)	6 (33.3)	
Associated ligament tear, no. (%)			
No tear	12 (92.3)	17 (94.4)	0.3484
LCL	1 (7.7)	0	
MCL	0	1 (5.6)	
IKDC^†^	84.3 ± 3.2	85.6 ± 4.4	0.3852
KOOS^‡^	78.0 ± 7.2	75 ± 7.4	0.2616
KOOS (sports)^§^	82.1 ± 5.8	84.8 ± 6.6	0.2446
Infection, no. (%)			
No	13 (100)	17 (94.4)	0.3884
Yes	0	1 (5.6)	
Pivot, no. (%)			
No	13 (100)	18 (100)	> 0.99
Yes	0	0	
Lachman, no. (%)			
No	7 (53.8)	11 (61.1)	0.6862
Yes	6 (46.2)	7 (38.9)	
Growth arrest, no. (%)			
No	13 (100)	17 (94.4)	0.3881
Yes	0	1 (5.6)	

All values are mean and standard deviations unless stated otherwise

*LCL* Lateral Collateral Ligament, *MCL* Medial Collateral Ligament, *IKDC* International Knee Documentation Committee, *KOOS* Knee injury and Osteoarthritis Outcome Score

^†^This score represents the 2000 International Knee Documentation Committee Subjective Knee Evaluation Form

^‡^This KOOS score relates to Knee-Related Quality of Life subscale

^§^This KOOS score relates to Sports and Recreation subscale

Mild and negligible valgus knee was found in the clinical examination of one case in the autograft group who underwent partial transphyseal ACL reconstruction. In the weight-bearing alignment view, the mechanical hip-knee-ankle (HKA) angle was measured on both lower limbs. The HKA angle on the treated side was 184° versus 180.5° contra-laterally. Alignment in the sagittal plane was unchanged. As the patient did not complain of pain, limping, and functional disturbance, no additional treatment was needed.

## Discussion

In here, we aimed to compare allografts and autografts in ACL reconstruction among a sample of adolescent athletes, in order to determine the graft of choice in this population. To the best of the authors’ knowledge, currently, no data exists on allograft ACL reconstruction in skeletally immature patients with joint laxity [22]. In here, for the first time, we found that allografts (in patients with joint laxity) and autografts did not differ regarding postoperative results during a 24-month follow-up period among skeletally immature athletes, although one case did present with septic arthritis in the autograft group.

In this age group, skeletal maturity is the most important determining factor as to which ACL reconstruction technique is selected. There is much concern regarding growth disturbance in younger patients due to their small knees, open physis, and the existence of long periods of remaining growth [23]. An anatomical study, utilizing three-dimensional MRI, showed that the volume of proximal tibial and distal femoral physis increases linearly with age. On the other hand, the volume percentage which is removed by drilling decreased linearly with age [24]. We used the Tanner staging system to evaluate skeletal maturity and to assess remaining growth, as it is among the most common systems used in the assessment of pediatric populations [16].

Current options for immature ACL reconstructions include physeal sparing procedures, transepiphyseal procedures, partial transphyseal procedures, and complete transphyseal procedures [10, 25].

The oblique nature of femoral tunnels in anatomical ACL reconstruction is in a way that most growth disturbances occur in the lateral distal femoral epiphysis [10, 14]. Therefore, partial transphyseal techniques have been introduced as a means of placing a more isometric tibial graft through a transphyseal tibial tunnel and avoiding the distal lateral femoral physis through transepiphyseal femoral tunnels [25]. In 2009, Henry et al. reported a case series of subacute ACL reconstructions with a transphyseal tibial tunnel and an anatomic epiphyseal femoral tunnel. In their study, no growth abnormalities were documented in patients [26]. In our study, patients with Tanner stage II–III (more than 1 year of growth remaining) were treated with partial transphyseal techniques to reconstruct an anatomical ACL with a minimum risk of growth arrest.

Complete transphyseal ACL reconstruction recommended for adolescents near skeletal maturity is familiar to most surgeons and allows more precise inside-out anatomic tunnel placement [10]. A number of case series have reported transphyseal ACL reconstructions using a variety of grafts and fixation devices with good outcomes in pediatric patients with little risk of growth disturbance [27, 28]. If some general rules, such as using soft tissue grafts with central and vertical tunnels related to the physis and avoiding over tensioning the grafts, are taken into consideration, the risk of growth disturbance will be reduced [25]. Although some studies recommend transphyseal ACL reconstruction for younger patients (Tanner II and III) [29], a recent meta-analysis found that the overall incidence of growth abnormalities in transphyseal ACL reconstructions was 2.1% [30]. So in our study, we applied this technique only for patients with Tanner stage VI (less than 1 year of growth remaining).

As mentioned before, much debate exists in literature regarding the appropriate graft for ACL reconstruction in adolescents [14, 31]. Most authors are unanimous that soft tissue grafts are less likely to create a bony bar and have minimal effect on growth [10, 25, 32]; however, controversy exists regarding the role of allograft in adolescent ACL reconstruction.

In a cohort study, Kaeding et al. attempted to identify predictors of outcome in ACL reconstruction. They found that the odds of graft rupture with an allograft reconstruction are four times higher than that of autograft reconstructions, especially in young and active patients [12].

In a recent study by Engelman et al. comparing survival of allograft and autograft ACL reconstruction in active adolescents, they found no differences regarding IKDC, Lysholm score, and rate of return to previous activity level between patients who had allograft and autografts; however, hazard ratio of graft failure was 4.4 times greater in the allograft group compared to the autograft group [13]. Although in our study, we did not find a difference regarding graft failure between the two graft types. This may be due to the fact that we used non-irradiated allograft as some studies have found that when comparing autografts and allografts, by excluding irradiated allografts, failure rates between autografts and allografts become insignificant [33].

In a prospective study by Edgar et al., autograft and allograft hamstring tendons for adult ACL reconstruction were compared. After a minimum of 3-year follow-up, they found no significant difference in functional score and laxity between the two groups. Furthermore, clinical outcome scores were similar between the groups, 3 to 6 years after reconstructive surgery [34].

On the one hand some concern does exist regarding the efficacy of allografts in patients with joint laxity, as one study found higher rates of anterior laxity in an adult group with allograft ACL reconstruction compared to an autograft groups [35], on the other hand, autografts in ACL reconstruction render higher rates of joint laxity among adolescents compared to adults [36]; thus, we selected allografts for ACL reconstruction in our adolescent group who had joint laxity and recurvatum. In our study, the results of allograft ACL reconstructions in adolescents with joint laxity were acceptable and comparable with autograft ACL reconstructions. The achieved mean 2000 IKDC subjective score and KOOS subscales in the allograft group were similar to that of the autograft group. After a 2-year follow-up, no case of obvious instability, giving way, locking, deep infection, and malalignment was documented in the allograft group. Some of the potential advantages of allografts, which include no donor-related morbidity, shorter operation time, and the ability to preoperatively select the appropriate length and diameter of graft, advocate the use allograft constructs in immature ACL reconstructions.

Despite numerous reports describing adolescent ACL reconstructions, no prospective, randomized studies were found comparing the clinical success of graft type, graft placement, or graft fixation in skeletally immature patients [10, 14, 37]. Existing studies have small sample sizes with a variety of outcome measures, making comparisons between studies difficult. Our study compared allografts and autografts using two different techniques for immature ACL reconstruction.

Although our study supports the use of allograft in adolescent ACL reconstruction, selection of type of graft should be done with consideration of multiple factors as both allografts and autograft have their own specific disadvantages. These include transmissible diseases, increased cost, tissue reaction, and late incorporation which are seen with allografts [38]; donor site morbidity, weakness, loss of knee flexion, and variability in graft sizes with hamstring tendon autografts [39, 40]; and weakness of quadricepts, patella fractures, and pain in the anterior of the knee seen with BPTB grafts (in adult ACL reconstruction) [39].

The study is not without limitations. Although we expected postoperative outcomes to be worse in the allograft group, considering that these individuals had associated joint laxity and recurvatum, our final results showed no significant difference between the two groups. Moreover, longer follow-up period may have rendered more accurate results. Mainly due to the nature of our study (rarity of our patients and specific inclusion criteria), we had a small sample size in both comparison groups, and although we did have a relatively long recruitment period of 6 years, we still had a low number of patients in our groups. This was further observed in our post hoc power analysis (which we performed) as we found a low power of study, which shows that perhaps the insignificant difference between the groups is most likely attributable to the low sample size; thus, interpretation of our results should be done with caution, and further studies with larger sample sizes and in the context of clinical trials that control any bias for comparison, between those who receive allografts and autografts in adolescent ACL reconstruction, are needed to support our findings. Moreover, considering that our study, to the best of the authors’ knowledge, is the first of its kind to report on the use of allografts in ACL reconstruction among skeletally immature patients with laxity, this renders our results invaluable. We used the Beighton score for measurement of laxity which is an all or none scale and does not provide any information regarding the degree of laxity. We found that the two grafts did not have any difference in follow-up studies, with one having the advantage among patients with joint laxity; however, we did not have any other previous report on the use of allografts among skeletally immature patients, which limited our ability to compare our results with that of other studies.

Among the strong points of the current study was that all patients were operated and then observed by a single surgeon, thus minimizing operator-related bias in the study.

## Conclusion

Considering the existing concern that joint laxity and recurvatum are among the precursors of non-comtact ACL injury in adolescents, bone-patellar-bone autografts are not applicable in this age group because of the open physis; moreover, considering that hamstring autografts are insufficient (size thickness and strechability), we recommend soft tissue allografts for ACL reconstruction in skeletally immature patients.
